# Exploring the molecular profile of localized colon cancer: insights from the AIO Colopredict Plus registry

**DOI:** 10.3389/fonc.2024.1434791

**Published:** 2024-11-19

**Authors:** Ira Ekmekciu, Doreen Maria Zucha, Jens Christmann, Sarah Wisser, Vera Heuer, Buelent Sargin, Stephan Hollerbach, Christof Lamberti, Lothar Müller, Celine Lugnier, Berlinda Verdoodt, Robin Denz, Tobias Terzer, Inke Feder, Anke Reinacher-Schick, Andrea Tannapfel, Iris Tischoff

**Affiliations:** ^1^ Department of Hematology, Oncology and Palliative Care, St. Josef Hospital, Ruhr University, Bochum, Germany; ^2^ Institute of Pathology, Ruhr University, Bochum, Germany; ^3^ Hematology and Medical Oncology, St-Marien-Hospital Lunen, Lunen, Germany; ^4^ Department of Gastroenterology, Allgemeines Krankenhaus (AKH) Celle, Celle, Germany; ^5^ Hematology and Oncology, Regiomed Hospital Group, Coburg, Germany; ^6^ Onkologie UnterEms, Leer, Germany; ^7^ Department of Medical Informatics, Biometrics and Epidemiology, Ruhr University, Bochum, Germany

**Keywords:** colon cancer, localized, real world data, microsatellite instability, RAS, BRAF

## Abstract

**Introduction:**

Understanding the mutational landscape of colon cancer (CC) is crucial for targeted therapy development. Microsatellite instability (MSI-H), rat sarcoma (RAS), and B-Raf proto-oncogene, serine/threonine kinase (BRAF) mutations (MT) are pivotal markers. Further investigation into clinicopathological features of RAS and BRAF MT in microsatellite stable (MSS) and MSI-H tumors is warranted.

**Methods:**

A retrospective analysis of 4883 localized CC patients (pts.) was conducted. Molecular profiling assessed MSI, KRAS, NRAS, and BRAF MT. Correlation with clinicopathological data employed ANOVA and Chi-square tests. Disease-free survival (DFS) and overall survival (OS) were analyzed adjusting for age, gender, sidedness, UICC stage, Charlson Comorbidity Index (CCI). A Cox model incorporated all variables as covariates.

**Results:**

This analysis included 4883 pts. (2302 female/2572 male, 3865 (79.2%) MSS, 1018 (20.8%) MSI-H). MSS pts. had more All-Wild Type (WT), KRAS MT, and NRAS MT tumors vs. MSI-H pts. (42.1% vs. 21.1%; 39.8% vs. 15.4%; 3.6% vs. 0.7%; p<0.001 for each). BRAF MT tumors (95.5% BRAF V600E MT) were more prevalent in MSI-H individuals (62.8% vs. 8.1%, p<0.001). KRAS and BRAF MT tumors were more frequently right-sided, while BRAF MT tumors were associated with female gender, advanced disease stage, lymph node positivity, and poorer differentiation in the MSS subset (p<0.001). Common KRAS mutations included p.G12D (30.44%) and p.G12V (21.3%) in MSS and p.G13D (28.9%) and p.G12D (22.37%) in MSI-H. NRAS MT tumors were dominated by codon 61 mutations (51.7%). Survival analysis revealed worst prognosis in BRAF MT MSS tumors (DFS: HR 1.74 (95% CI 1.15-2.62, p=0.009; OS: HR 1.61 (95% CI 0.99-2.6), p=0.055). The 3-years DFS and 5-years OS rates were lowest in this subset (61.6% and 57.7% respectively).

**Discussion:**

These findings highlight the complex interplay between molecular subtypes, clinicopathological features, and survival outcomes in early CC. Further research is needed to elucidate underlying mechanisms and develop personalized treatment strategies.

## 1 Introduction

Colon cancer (CC) remains a significant public health concern worldwide, representing a leading cause of cancer-related morbidity and mortality. Despite advancements in diagnosis and treatment, the heterogeneous nature of CC poses challenges for clinicians in predicting patient outcomes and optimizing therapeutic strategies. For stage III and high risk stage II disease adjuvant chemotherapy is recommended using fluopyrimidins +/- oxaliplatin (1, 2). However, the benefit from adjuvant chemotherapy in these patients (pts) appears to be relatively modest, ranging from 5% to 20% (3).

Recent studies have underscored the significant role of molecular changes, particularly mutations in key genes such as those associated with deficient mismatch repair protein (dMMR) leading to microsatellite instability-high status (MSI-H), the rat sarcoma (RAS) family (including KRAS/NRAS/HRAS), and the B-Raf proto-oncogene, serine/threonine kinase (BRAF) in shaping the pathogenesis and clinical behavior of CC. Evaluation of mutational status, especially in metastatic disease, is strongly recommended in all guidelines (4). The development of targeted therapy approaches, particularly for pts with MSI-H and BRAF-V600E mutations, evaluated in clinical trials, has led to more personalized treatment options for these subgroups in metastatic CC (5, 6).

Understanding the interplay between these molecular subtypes and their implications for patient prognosis and treatment response is crucial for advancing personalized medicine approaches in CC management. Activating mutations in KRAS and NRAS are recognized as negative predictors for anti-epidermal growth factor receptor (anti-EGFR) treatment (7, 8). Moreover, targetable KRAS submutations have emerged. Compounds like sotorasib and adagrasib, which selectively inhibit the KRAS p.G12C mutation, are being tested in combination with anti-EGFR antibodies in the context of metastatic CC and have delivered promising results (9, 10).

While these investigations predominantly focus on advanced CC, clinical and molecular data regarding localized disease are less common. In a previous study, we analyzed the AIO Colopredict Plus (CPP) registry, specifically focusing on elderly individuals with CC, and found that adjuvant chemotherapy is an independent positive prognostic factor for overall survival (OS) (11, 12). Furthermore, we identified a subset of elderly pts with fewer comorbidities who may particularly benefit from adjuvant chemotherapy.

In this study, we conducted a comprehensive analysis of the genetic landscape of CC using real-world data from our CPP registry spanning over a decade. Using advanced sequencing methods, we aimed to elucidate the prevalence and clinical significance of MSI, RAS, and BRAF mutations in nonmetastatic CC pts. The primary objective was to investigate the role of RAS and BRAF mutations in correlation with MSI status and associated clinicopathological characteristics. Our findings provide insights into the complex molecular landscape of CC and offer potential implications for pts stratification and treatment decision-making. Through this research, we seek to contribute to the ongoing efforts to improve outcomes for CC pts by uncovering new avenues for targeted therapy and personalized care.

## 2 Material and methods

In September 2013, the CPP molecular registry study commenced in 20 German community cancer centers, subsequently expanding to encompass over 200 German CC centers. All study participants provided informed consent, and the study protocol was approved by the Ethics Committee of the Ruhr-University Bochum (registration number: 17-6151_13 and 12-4449; DRKS-ID: DRKS00004305). The study gathered demographic data including age, sex, body mass index (BMI), as well as comorbidities, tumor localization and survival status. Newly diagnosed CC cases with histopathological confirmation at Union Internationale Contre le Cancer (UICC) stages II and III were initially enrolled, with the cohort later expanded in 2018 to include UICC stage I cases. Tumors were classified according to the current WHO 2019 guidelines and the TNM system outlined in the American Joint Committee on Cancer (AJCC) Cancer Staging Manual, Eighth Edition. Cases of UICC stage IV CC and rectal cancer were excluded from the study. The data collection cutoff point was February, 2023.

### 2.1 Tissue analysis by next generation sequencing (NGS)

Molecular analysis was performed centrally at the Institute of Pathology of the Ruhr University Bochum. DNA concentration was measured by QuantiFluor ^®^ ONE dsDNA on the Quantus (Promega Corporation, Madison, USA). All further concentration measurements were performed using QubitTM dsDNA HS. According to the manufacturer’s instructions, the QIAGEN Colorectal Cancer Panel (QIAGEN GmbH, Hilden, GER) was used to amplify target regions of 72 CC related genes (**Supplementary Table 1**), among others BRAF and RAS (K- and N-RAS). Libraries were prepared with 50 ng of genomic DNA. Sequencing of the final libraries was performed using NextSeq 550 Illumina sequencer (Illumina Inc., San Diego, CA). For data analysis the QIAGEN CLC Genomics Workbench (Version 21) was used. Analysis and assessment of detected variants was performed using IGV, MutationTaster, ClinVar, Cosmic, and Qiagen Clinical Insight. Only disease-causing mutations (Variant Allele Frequency (VAF) ≥ 5%, read coverage ≥100 unique reads mean coverage in min. 80% of sequenced nucleotides, read balance ≥0,2) were reported. In individual cases, hand-curated variants in known driver genes were reported with slightly worse quality parameters if they were classified as true according to expert opinion. Data concerning RAS, BRAF and MSI were extracted from NGS data.

### 2.2 Detection of MSI status

MSI-testing was performed using both immunohistochemistry for four proteins (MLH1, MLH6, MSH2, PMS2) and PCR-based fragment-length analysis as previously described (11). In cases of inconsistent results, NGS was employed as the gold standard. A discrepancy between immunohistochemistry and PCR-based analysis was observed in 12% of cases (11).

For the detection of MSI status via NGS, eight well-established MSI loci (BAT25, BAT26, D2S123, NR21, NR22, NR24, D5S346, D17S250) were sequenced and the respective reads were compared with those of a validated, non-tumor tissue baseline in the same entity. By analyzing the length distribution histogram, each sample was compared with the baseline to determine the MSI status.

### 2.3 Statistics

Arithmetic means and standard deviations were computed for continuous variables, while frequencies and percentages were calculated for categorical variables. ANOVA tests and Chi-Square Tests were used to test whether these variables were distributed differently in the relevant subgroups for continuous variables and categorical variables respectively. OS was defined as the time until death from any cause, with pts lost to follow-up or still alive at the end of the study period being censored. Disease-Free Survival (DFS) combined both relapse and death endpoints. Confounder-adjusted survival curves were generated using G-computation based on Cox regression models (13) and hazard ratios (HRs) with 95% confidence intervals (CIs) were calculated. Adjusting factors included age, sex, Charlson Comorbidity Index (CCI), UICC stage, and tumor location (sidedness). Statistical significance was defined as p < 0.05. All statistical analysis were carried out using R (version 4.2.1).

## 3 Results

### 3.1 Study population

**Figure 1:**
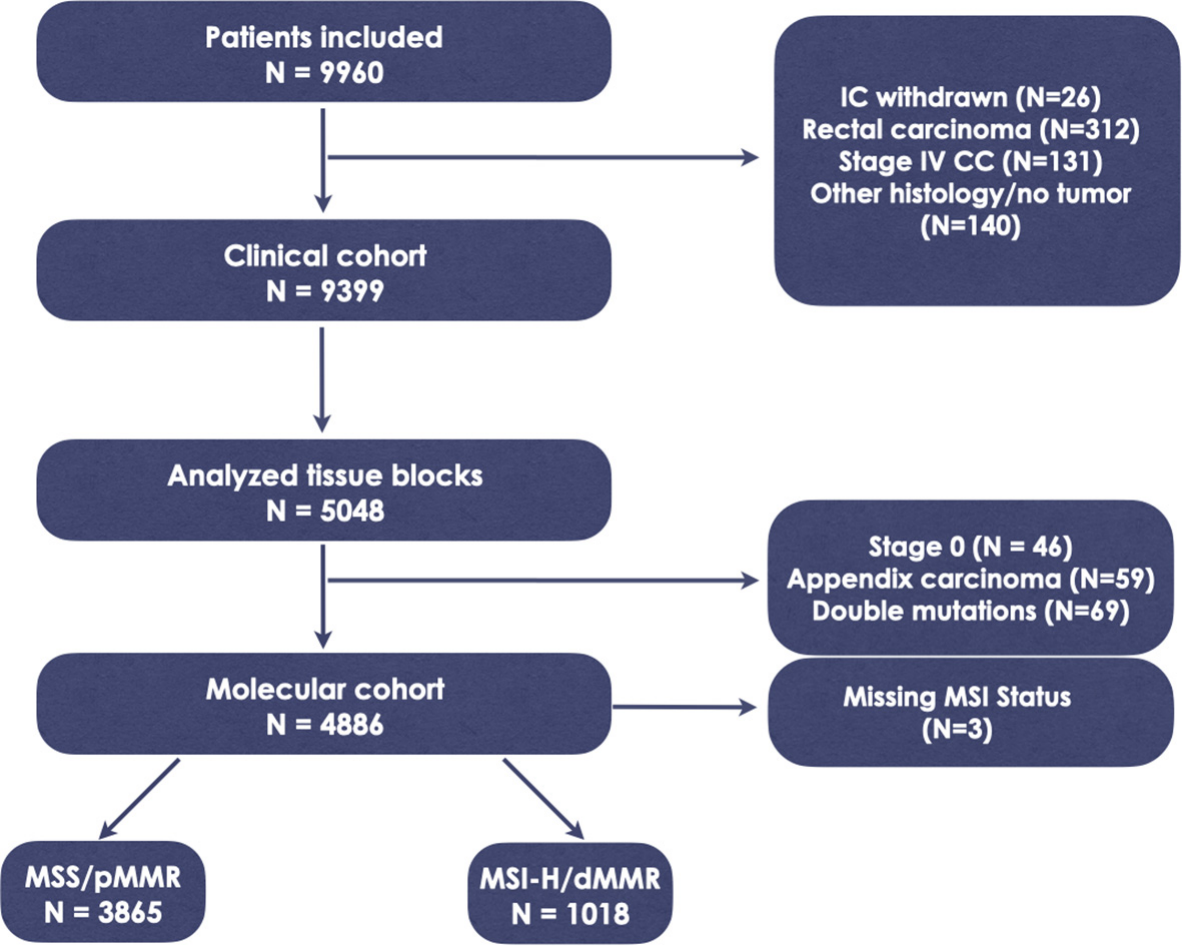
Study flowchart. IC, informed consent; CC, colon cancer; MSS, microsatellite stability; MSI-H, microsatellite instability; dMMR, deficient mismatch repair protein; pMMR, proficient mismatch repair protein.

Between September 2013 and February 2023, a total of 9960 pts. were enrolled in CPP. Following the exclusion of 561 patients who did not meet the inclusion criteria (**Figure 1**), 9399 patients remained eligible for analysis. Histological analysis and NGS was applied to analyze adequate tissue samples from 5048 randomly selected cases, representing 50.7% of the total cases, up to the cutoff date of February 2023. After excluding 165 cases due to Stage 0, histology indicating appendix carcinoma, missing MSI data, or the presence of double mutations, the final molecular cohort for this analysis comprised 4883 patients. Among these, 3865 individuals (79.2%) were microsatellite stable (MSS), while 1018 (20.8%) were microsatellite instable (MSI-H) (**Figure 1**). The study population consisted of 2302 females and 2572 males. Baseline characteristics of the entire study population, correlated with the mutational status of KRAS, BRAF, and NRAS, are detailed in **Supplementary Table 2**. The mean follow-up duration was 29.5 months.

### 3.2 Baseline clinical, histopathological and molecular characteristics of MSS and MSI-H cohort

**Figure 2:**
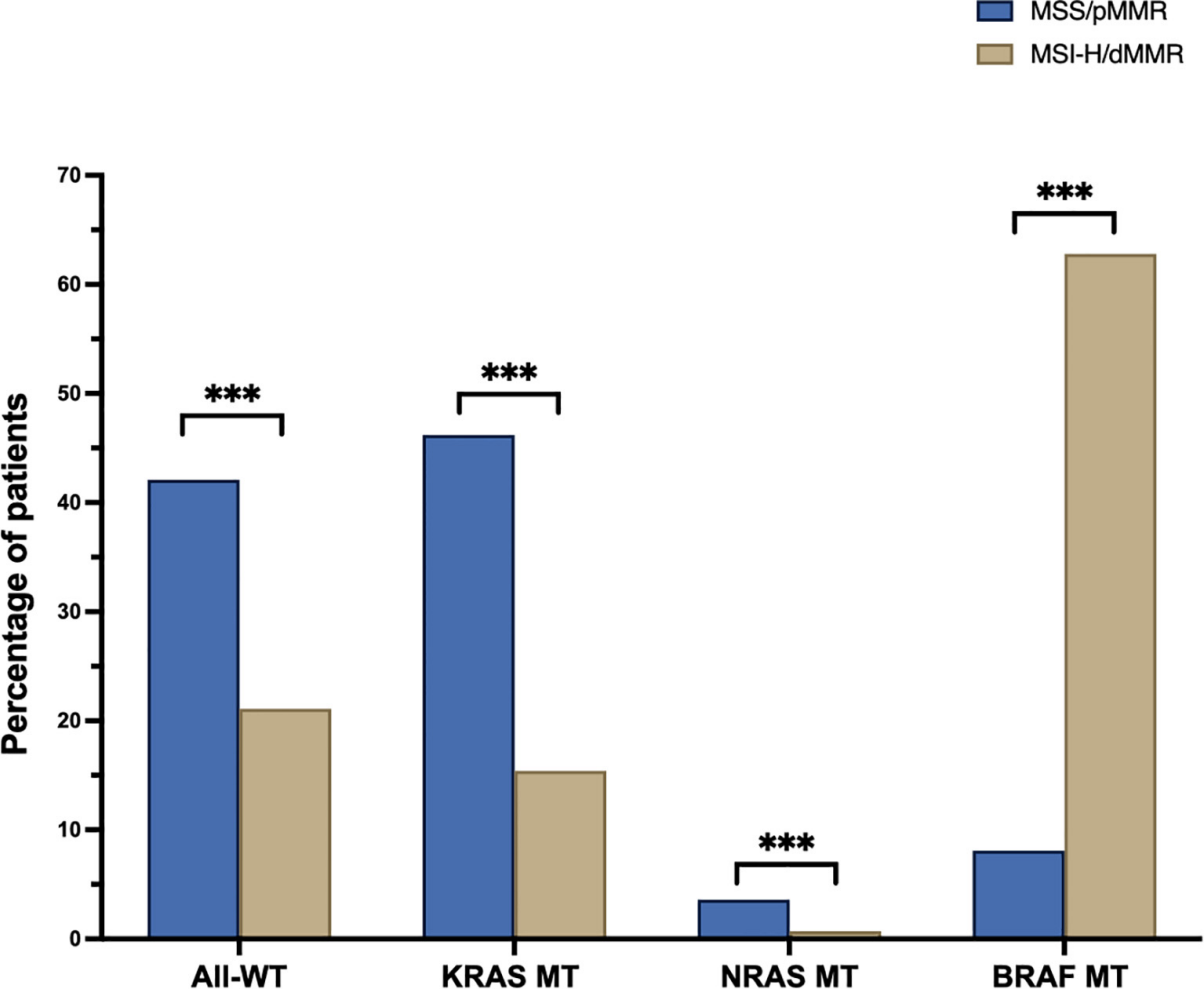
Percentage of wildtype (All-WT) and mutated (MT) pts. in the microsatellite stable (MSS, blue) and instable (MSI-H, sand) cohorts (BRAF, B-Raf proto-oncogene, serine/threonine kinase; KRAS, Kirsten Rat Sarcoma; NRAS, Neuroblastoma ras); ***p < 0.001.

Across the entire study cohort, 37.7% were identified as having All-Wild Type (WT), 39.8% had KRAS mutated (MT) tumors, 3% exhibited NRAS MT, and 19.5% displayed BRAF MT tumors (**Supplementary Table 2**). In the MSS cohort, the prevalence rate was 42.1% All-WT, 46.2% KRAS MT, 3.6% NRAS MT and 8.1% BRAF MT, respectively. BRAF MT tumors were significantly more prevalent in the MSI-H population, constituting 62.8% of this subgroup (p<0.001), while All-WT, KRAS MT, and NRAS MT cancer specimens were less frequent, accounting for 21.1%, 15.4%, and 0.7%, respectively (**Figure 2**, p<0.001 for each).

In the MSS population, All-WT as well as KRAS and NRAS MT tumors were more often found in males, while tumors bearing mutations in the BRAF gene were more commonly identified in female pts. All MT tumors exhibited a higher likelihood of presenting with lymph node positivity and were associated with more advanced disease stages at time of diagnosis, particularly in BRAF MT specimens. Notably, both KRAS- and BRAF-MT tumors demonstrated a predilection for localization in the right colon. Furthermore, BRAF mutations correlated with poorer differentiation and the presence of more aggressive histological subtypes, including mucinous adenocarcinoma (**Table 1**).

**Table 1:** Clinical and histopathological baseline characteristics of molecular subgroups in the MSS and MSI- H cohort.

Characteristic	MSS/pMMR				p-Value	MSI-H/dMMR				p-Value
	All-WT (N=1627)	KRAS MT (N=1787)	NRAS MT (N=139)	BRAF MT (N=312)		All-WT (N=215)	KRAS MT (N=157)	NRAS MT (N=7)	BRAF MT (N=639)	
**Age (in years)**					0.39					**<0.001**
Median	71	69	59	70		73	69	59	70	
Mean (SD)	69.1 (11.9)	69.7 (11.5)	68.6 (12.4)	68.9 (11.2)		69.8 (13.1)	65.9 (14.2)	62.7 (16.7)	76 (8.9)	
Range	25 - 95	30 - 96	26 - 92	29 - 92		27- 93	27 - 94	44 - 84	41 - 100	
**Age (categories)**					0.12					**<0.001**
< 50	107 (6.6%)	81 (4.5%)	9 (6.5%)	13 (4.2%)		20 (9.3%)	22 (14.0%)	2 (28.6%)	3 (0.5%)	
50 -70	630 (38.7%)	723 (40.5%)	55 (39.6%)	137 (43.9%)		74 (34.4%)	60 (38.2%)	2 (28.6%)	133 (20.8%)	
≥ 70	890 (54.7%)	983 (55.0%)	75 (54.0%)	162 (51.9%)		121 (56.3%)	75 (47.8%)	3 (42.9%)	503 (78.7%)	
**Sex**					**<0.001**					**<0.001**
Female	591 (36.4%)	795 (44.6%)	63 (45.3%)	163 (52.2%)		133 (62.7%)	74 (47.1%)	3 (42.9%)	479 (75.1%)	
Male	1032 (63.6%)	988 (55.4%)	76 (54.7%)	149 (47.8%)		79 (37.3%)	83 (52.9%)	4 (57.1%)	159 (24.9%)	
Missing	4	4	0	0		3	0	0	1	
**CCI**					0.52					0.75
< 5	1605 (98.6%)	1767 (98.9%)	139 (100.0%)	309 (99.0%)		213 (99.1%)	156 (99.4%)	7 (100.0%)	629 (98.4%)	
≥ 5	22 (1.4%)	20 (1.1%)	0 (0.0%)	3 (1.0%)		2 (0.9%)	1 (0.6%)	0 (0.0%)	10 (1.6%)	
**BMI**					0.11					0.98
Mean (SD)	27.1 (5.2)	26.7 (5.0)	26.5 (4.7)	27.1 (5.3)		26.445 (5.129)	26.429 (4.566)	27.122 (4.926)	26.352 (4.973)	
Range	12.7- 57.5	14.5 - 54.2	15.9- 42.2	15.2- 52.5		16.420 - 45.280	16.670 - 45.170	21.230 - 33.910	13.840 - 48.070	
Missing	121	150	7	23		19	19	1	69	
**BMI (categories)**					0.28					0.71
< 18.5	37 (2.5%)	42 (2.6%)	5 (3.8%)	8 (2.8%)		10 (5.1%)	3 (2.2%)	0 (0.0%)	16 (2.8%)	
18.5 - 25	528 (35.1%)	638 (39.0%)	44 (33.3%)	100 (34.6%)		74 (37.8%)	51 (37.0%)	3 (50.0%)	221 (38.8%)	
≥ 25	941 (62.5%)	957 (58.5%)	83 (62.9%)	181 (62.6%)		112 (57.1%)	84 (60.9%)	3 (50.0%)	333 (58.4%)	
Missing	121	150	7	23		19	19	1	69	
**T-Stage**					0.55					0.29
T 1/2	252 (15.5%)	269 (15.1%)	16 (11.5%)	52 (16.7%)		31 (14.4%)	16 (10.2%)	0 (0.0%)	97 (15.2%)	
T 3/4	1375 (84.5%)	1518 (84.9%)	123 (88.5%)	260 (83.3%)		184 (85.6%)	141 (89.8%)	7 (100.0%)	542 (84.8%)	
**N-Stage**					**<0.001**					0.053
N0	985 (60.8%)	1009 (56.7%)	77 (55.4%)	133 (43.2%)		135 (63.1%)	117 (75.0%)	5 (71.4%)	406 (63.8%)	
N+	635 (39.2%)	771 (43.3%)	62 (44.6%)	175 (56.8%)		79 (36.9%)	39 (25.0%)	2 (28.6%)	230 (36.2%)	
**UICC**					**<0.001**					**0.011**
I	178 (10.9%)	172 (9.6%)	7 (5.0%)	29 (9.3%)		23 (10.7%)	10 (6.4%)	0 (0.0%)	71 (11.1%)	
II	806 (49.6%)	841 (47.1%)	71 (51.1%)	107 (34.3%)		112 (52.1%)	108 (68.8%)	5 (71.4%)	331 (51.8%)	
III	642 (39.5%)	774 (43.3%)	61 (43.9%)	176 (56.4%)		80 (37.2%)	39 (24.8%)	2 (28.6%)	237 (37.1%)	
**Differential Grade**					**<0.001**					
G 1/2	1372 (84.3%)	1493 (83.5%)	118 (84.9%)	211 (67.6%)		113 (52.6%)	108 (68.8%)	4 (57.1%)	324 (50.7%)	
G 3/4	255 (15.7%)	294 (16.5%)	21 (15.1%)	101 (32.4%)		102 (47.4%)	49 (31.2%)	3 (42.9%)	315 (49.3%)	
**Histological Subtype**					**<0.001**					0.78
Adenocarcinoma	1535 (96.3%)	1664 (95.0%)	132 (96.4%)	279 (90.6%)		185 (89.4%)	140 (91.5%)	6 (85.7%)	545 (86.5%)	
Mucinous adenocarcinoma	29 (1.8%)	66 (3.8%)	5 (3.6%)	24 (7.8%)		15 (7.2%)	11 (7.2%)	1 (14.3%)	56 (8.9%)	
Signet-ring cell carcinoma	6 (0.4%)	0 (0.0%)	0 (0.0%)	1 (0.3%)		1 (0.5%)	0 (0.0%)	0 (0.0%)	3 (0.5%)	
Others	24 (1.5%)	22 (1.3%)	0 (0.0%)	4 (1.3%)		6 (2.9%)	2 (1.3%)	0 (0.0%)	26 (4.1%)	
Missing	33	35	2	4		8	4	0	9	
**Chemotherapy**					0.092					0.55
Yes	586 (40.4%)	684 (43.0%)	52 (44.1%)	133 (48.2%)		63 (32.1%)	37 (27.2%)	1 (20.0%)	155 (27.1%)	
No	863 (59.6%)	906 (57.0%)	66 (55.9%)	143 (51.8%)		133 (67.9%)	99 (72.8%)	4 (80.0%)	418 (72.9%)	
Missing	178	197	21	36		19	21	2	66	
**Localization**					**<0.001**					**<0.001**
Cecum	112 (7.0%)	381 (21.7%)	21 (15.2%)	46 (14.9%)		37 (17.3%)	30 (19.5%)	1 (14.3%)	160 (25.2%)	
Ascending colon	260 (16.2%)	452 (25.7%)	32 (23.2%)	115 (37.2%)		75 (35.0%)	51 (33.1%)	2 (28.6%)	286 (45.0%)	
Hepatic flexure	90 (5.6%)	98 (5.6%)	5 (3.6%)	34 (11.0%)		24 (11.2%)	11 (7.1%)	0 (0.0%)	66 (10.4%)	
Transverse colon	160 (10.0%)	138 (7.8%)	8 (5.8%)	38 (12.3%)		34 (15.9%)	16 (10.4%)	3 (42.9%)	68 (10.7%)	
Splenic flexure	64 (4.0%)	76 (4.3%)	7 (5.1%)	12 (3.9%)		6 (2.8%)	10 (6.5%)	0 (0.0%)	14 (2.2%)	
Descending colon	126 (7.9%)	86 (4.9%)	10 (7.2%)	17 (5.5%)		12 (5.6%)	11 (7.1%)	1 (14.3%)	18 (2.8%)	
Sigmoid colon	736 (45.9%)	494 (28.1%)	50 (36.2%)	45 (14.6%)		25 (11.7%)	25 (16.2%)	0 (0.0%)	23 (3.6%)	
Rectosigmoid junction	57 (3.6%)	33 (1.9%)	5 (3.6%)	2 (0.6%)		1 (0.5%)	0 (0.0%)	0 (0.0%)	1 (0.2%)	
Missing	22	29	1	3		1	3	0	3	
**Sidedness**					**<0.001**					**<0.001**
Right	622 (38.8%)	1069 (60.8%)	66 (47.8%)	233 (75.4%)		170 (79.4%)	108 (70.1%)	6 (85.7%)	580 (91.2%)	
Left	983 (61.2%)	689 (39.2%)	72 (52.2%)	76 (24.6%)		44 (20.6%)	46 (29.9%)	1 (14.3%)	56 (8.8%)	
Missing	22	29	1	3		1	3	0	3	

BMI, Body Mass Index; BRAF MT, mutated for B-Raf proto-oncogene, serine/threonine kinase; dMMR, deficient mismatch repair; KRAS MT, mutated for Kirsten Rat Sarcoma; MSI-H, microsatellite instability; MSS, microsatellite stability; NRAS MT, mutated for Neuroblastoma ras; pMMR, proficient mismatch repair protein; SD, Standard deviation; WT, Wild Type. Statistically significant values have been highlighted in bold

4Among pts. with MSI-H tumors, BRAF mutations were more frequently observed in elderly and female individuals. Additionally, all MSI-H tumors were predominantly localized in the right colon, with this tendency being particularly pronounced in BRAF MT tumors, with 91.2% demonstrating right-sidedness. Conversely, KRAS and NRAS MT MSI-H tumors were more commonly found in younger patients, males, and those with early-stage disease (UICC I-II), often displaying better histological differentiation (**Table 1**).

In addition, we evaluated the distribution of mutations of the four MMR genes (MLH1, MSH2, PMS2, and MSH6) in MSI-H tumors. In the BRAF wild-type population, the most frequent MMR gene mutation was in MLH1 (22%), followed by MSH6 (21.6%), MSH2 (18.9%), and PMS2 (15.8%). In contrast, the most common MMR gene alteration in the BRAF V600 mutated population was in MSH6 (17.7%), with frequencies of 8.3%, 6.3%, and 9.7% for MLH1, MSH2, and PMS2, respectively (**Supplementary Table 4**).

### 3.3 RAS hotspot mutations

In the analysis of individual KRAS hotspot mutations, 62 patients with double mutations in the KRAS gene were excluded and will be described elsewhere. **Supplementary Table 3** illustrates the correlations of individual KRAS hotspot mutations with clinical and histopathological characteristics for both the MSS and MSI-H cohorts.

In both cohorts, all KRAS mutations (excluding p.G12D in the MSI-H cohort) were predominantly localized in the right colon. No significant differences were observed regarding age, UICC Stage, or histopathological subtype. In the MSI-H population, mutations such as p.G12D, p.G13D, and p.G12A were more prevalent in males, while p.A146T and p.G12C were more common in females, although the overall numbers were low.

**Figure 3:**
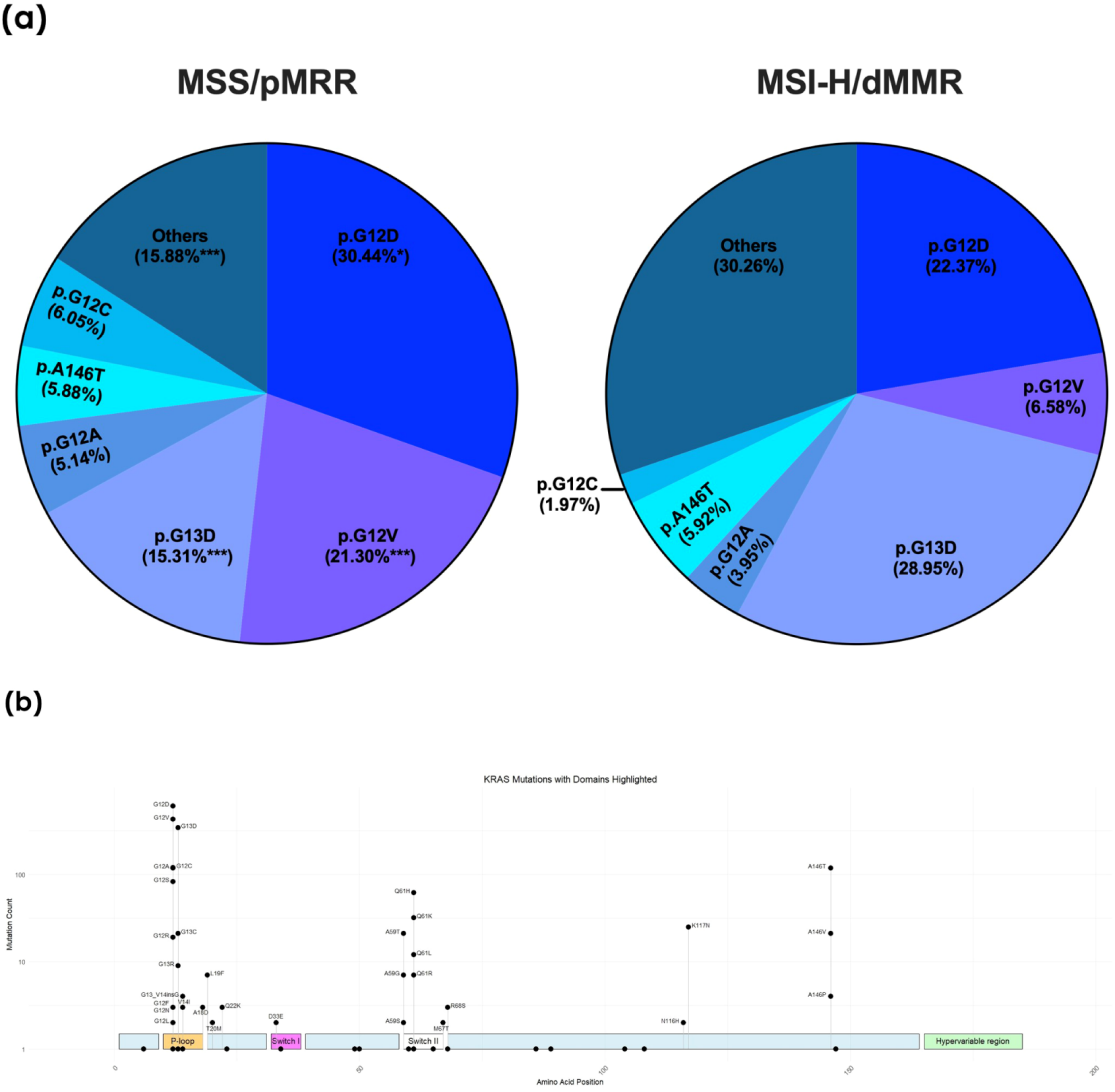
**(A)** Frequency of individual KRAS hotspot mutations in the microsatellite stable (MSS) and instable (MSI-H) cohorts. dMMR, deficient mismatch repair protein; pMMR, proficient mismatch repair protein: **p < 0.01; ***p < 0.001. **(B)** Distribution of the individual KRAS mutations with highlighted domains of the KRAS gene. The height of the respective point corresponds to the frequency of the mutation found in the collective.

Among the 1751 KRAS-mutated MSS patients, the distribution of mutations was as follows: 30.44% p.G12D, 21.3% p.G12V, 15.31% p.G13D, 5.14% p.G12A, 5.88% p.A146T, and 6.05% p.G12C, with 15.88% classified as other mutations (**Figure 3A**). The majority of the mutations were located in the known hotspot regions of the RAS gene (**Figure 3B**). Notably, the most frequent KRAS mutation in the MSI-H cohort was p.G13D, comprising 28.9% of all KRAS mutations. Codon 12 mutations were less prevalent in the MSI-H population (p.G12D 22.37%, p.G12V 6.58%, p.G12A 3.95%, and p.G12C 1.97%), while there were no significant differences in the frequency of the p.A146T mutation between the MSS and MSI-H cohorts (5.88% vs. 5.92%, respectively). The MSI-H population exhibited a greater diversity of KRAS mutations, including rarer and uncommon mutations, categorized as “others” in this analysis (30.26% in the MSI-H vs. 15.88% in the MSS population) (**Figure 3A**).

In the overall population, 146 (3%) NRAS mutations were detected. Three pts. were excluded due to double mutations. The most prevalent NRAS mutations consisted of codon 61 mutations, with p.Q61K observed in 34 out of 143 cases (23.77%), p.Q61R in 22 out of 143 cases (15.38%), and p.Q61L in 14 out of 143 cases (9.79%). Following these, codon 12 mutations were identified, with p.G12D occurring in 30 out of 143 cases (20.98%) and p.G12C in 6 out of 143 cases (6.29%). Of the NRAS-mutated patients, 136 out of 143 (95.1%) belonged to the MSS cohort, indicating a rare co-occurrence of NRAS mutations and MSI-H status (data not shown).

### 3.4 BRAF mutations

In the cohort of 951 BRAF MT tumors, only 4 possessed double mutations and were thus excluded from this subgroup analysis. Notably, the majority of BRAF MT tumors also demonstrated MSI-H status (636/947, 67.16%). Furthermore, the overwhelming majority of BRAF mutations in both the MSS and MSI subgroups were BRAF V600E mutations, accounting for 89% in the MSS cohort and 98.6% in the MSI-H cohort, respectively (**Table 2**). BRAF-V600E MT MSS tumors were more frequently associated with patients with a higher BMI and exhibited poorer differentiation compared to Non-V600E mutated tumors. Interestingly, BRAF-V600E mutated MSI-H patients were the oldest, with a median age of 77 years. Moreover, BRAF MT tumors, both in the MSS and MSI-H groups, were predominantly localized in the right colon. Specifically, only 24.1% of BRAF MT MSS tumors and 8.8% of BRAF MT MSI-H tumors were localized in the left colon (**Table 2**).

**Table 2:** Clinical and histopathological baseline characteristics of BRAF V600E and Non-V600E in the MSS and MSI- H cohort.

Characteristic	BRAF MT MSS/pMMR		p-Value	BRAF MT MSI/dMMR		p-Value
	V600E (N=277)	Non-V600E (N=34)		V600E (N=627)	Non-V600E (N=9)	
**Age (in years)**			0.27			**<0.001**
Median	70	71		77	74	
Mean (SD)	69.1 (11.1)	66.9 (12.5)		76.2 (8.7)	66 (14.8)	
Range	29 - 92	33 - 86		41 - 100	43 - 83	
**Age (categories)**			0.32			**<0.001**
< 50	10 (3.6%)	3 (8.8%)		2 (0.3%)	1 (11.1%)	
50 -70	124 (44.8%)	13 (38.2%)		130 (20.7%)	3 (33.3%)	
≥ 70	143 (51.6%)	18 (52.9%)		495 (78.9%)	5 (55.6%)	
**Sex**			0.51			0.17
Female	147 (53.1%)	16 (47.1%)		472 (75.4%)	5 (55.6%)	
Male	130 (46.9%)	18 (52.9%)		154 (24.6%)	4 (44.4%)	
Missing				1	0	
**CCI**			0.54			0.70
< 5	274 (98.9%)	34 (100.0%)		617 (98.4%)	9 (100.0%)	
≥ 5	3 (1.1%)	0 (0.0%)		10 (1.6%)	0 (0.0%)	
**BMI**			0.33			0.97
Mean (SD)	27.2 (5.2)	26.2 (5.7)		26.4 (5.0)	26.3 (3.8)	
Range	15.2- 52.5	17.2 - 37.1		13.8 - 48.0	19.8 - 30.4	
Missing	19	4				
**BMI (categories)**			**0.033**			0.88
< 18.5	5 (1.9%)	3 (10.0%)		16 (2.9%)	0 (0.0%)	
18.5 - 25	88 (34.1%)	11 (36.7%)		216 (38.6%)	3 (37.5%)	
≥ 25	165 (64.0%)	16 (53.3%)		328 (58.6%)	5 (62.5%)	
Missing	19	4				
**T-Stage**			0.48			0.74
T 1/2	44 (15.9%)	7 (20.6%)		95 (15.2%)	1 (11.1%)	
T 3/4	233 (84.1%)	27 (79.4%)		532 (84.8%)	8 (88.9%)	
**N-Stage**			0.91			0.38
N0	119 (43.4%)	14 (42.4%)		398 (63.8%)	7 (77.8%)	
N+	155 (56.6%)	19 (57.6%)		226 (36.2%)	2 (22.2%)	
**UICC**			0.17			
I	23 (8.3%)	6 (17.6%)		69 (11.0%)	1 (11.1%)	0.97
II	98 (35.4%)	9 (26.5%)		326 (52.0%)	5 (55.6%)	
III	156 (56.3%)	19 (55.9%)		232 (37.0%)	3 (33.3%)	
**Differential Grade**			**0.021**			0.34
G 1/2	182 (65.7%)	29 (85.3%)		317 (50.6%)	6 (66.7%)	
G 3/4	95 (34.3%)	5 (14.7%)		310 (49.4%)	3 (33.3%)	
**Histological Subtype**			0.26			0.70
Adenocarcinoma	244 (89.4%)	34 (100.0%)		533 (86.2%)	9 (100.0%)	
Mucinous adenocarcinoma	24 (8.8%)	0 (0.0%)		56 (9.1%)	0 (0.0%)	
Others	5 (1.9%)	0 (0.0%)		29 (4.7%)	0 (0.0%)	
Missing	4	0		9	0	
**Chemotherapy**			0.67			0.51
Yes	116 (47.5%)	16 (51.6%)		152 (27.0%)	3 (37.5%)	
No	128 (52.5%)	15 (48.4%)		410 (73.0%)	5 (62.5%)	
Missing				65	1	
**Localization**			**<0.001**			0.41
Cecum	41 (15.0%)	5 (14.7%)		157 (25.2%)	3 (33.3%)	
Ascending colon	110 (40.1%)	5 (14.7%)		281 (45.0%)	2 (22.2%)	
Hepatic flexure	32 (11.7%)	2 (5.9%)		66 (10.6%)	0 (0.0%)	
Transverse colon	33 (12.0%)	5 (14.7%)		66 (10.6%)	2 (22.2%)	
Splenic flexure	11 (4.0%)	1 (2.9%)		14 (2.2%)	0 (0.0%)	
Descending colon	16 (5.8%)	1 (2.9%)		17 (2.7%)	1 (11.1%)	
Sigmoid colon	31 (11.3%)	13 (38.2%)		22 (3.5%)	1 (11.1%)	
Rectosigmoid junction	0 (0.0%)	2 (5.9%)		1 (0.2%)	0 (0.0%)	
Missing	3	0		3	0	
**Sidedness**			**<0.001**			0.16
Right	216 (78.8%)	17 (50.0%)		570 (91.3%)	7 (77.8%)	
Left	58 (21.2%)	17 (50.0%)		54 (8.7%)	2 (22.2%)	
Missing	3	0		3	0	

BMI, Body Mass Index; BRAF MT, mutated for B-Raf proto-oncogene, serine/threonine kinase; dMMR, deficient mismatch repair; MSI-H, microsatellite instability; MSS, microsatellite stability; pMMR, proficient mismatch repair protein; SD, Standard deviation. Statistically significant values have been highlighted in bold

### 3.5 Survival

Due to the limited number of NRAS MT cases in the overall population, survival analysis combined KRAS and NRAS MT tumors into the RAS MT category. Cox proportional hazards regressions were conducted to assess both the main effects of MSI status and mutation group, as well as their interaction effects. No significant impact on survival was observed for RAS MT tumors in either MSS or MSI-H subsets. However, in the MSS cohort, BRAF MT tumors exhibited a significantly worse DFS (HR=1.74, 95% CI: 1.15-2.62, p=0.009). Although this subgroup showed similar effects for OS, statistical significance was not reached (HR 1.6, 95% CI: 0.99-2.60, p=0.055). Conversely, the occurrence of BRAF MT did not significantly influence survival in the MSI-H subset.

**Figure 4:**
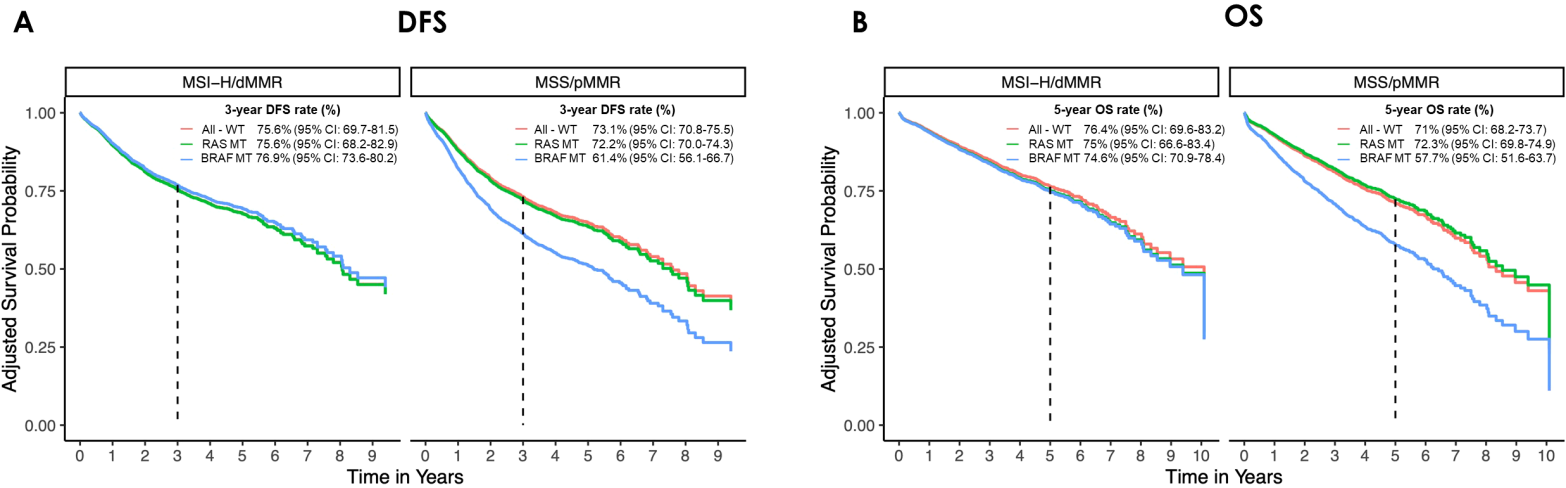
Adjusted survival curves for **(A)** disease-free survival (DFS) and **(B)** overall survival (OS) in the microsatellite stable (MSS/pMMR) and instable (MSI-H/dMMR) cohorts by mutational status. The curves have been adjusted for age, gender, UICC stage, sidedness, CCI. (All-WT, wild type tumors; BRAF MT, mutated for B-Raf proto-oncogene, serine/threonine kinase; CI, confidence interval; dMMR: deficient mismatch repair protein; pMMR, proficient mismatch repair protein; RAS MT, mutated for rat sarcoma).

Adjusted survival probabilities for DFS and OS are presented in **Figure 4**. In the MSS cohort, the 3-year DFS rates were 73.1% for All-WT (95% CI: 70.8-75.5), 72.2% for RAS MT (95% CI: 70.0-74.3), and 61.4% for BRAF MT (95% CI: 56.1-66.7) pts respectively. The corresponding 5-year OS survival rates were 71% (95% CI: 68.2-73.7), 72.3% (95% CI: 69.8-74.9), and 57.7% (95% CI: 51.6-63.7). For All-WT, RAS MT, and BRAF MT MSI-H tumors the 3-year DFS rate was 75.6% (95% CI: 69.7-81.5), 75.6% (95% CI: 68.2-82.9), and 76.9% (95% CI: 73.6-80.2) with corresponding 5-year OS rates of 76.4% (95% CI: 69.6-83.2), 75% (95% CI: 66.6-83.4), and 74.6% (95% CI: 70.9-78.4).

## 4 Discussion

The findings of this study shed light on the prevalence and clinical implications of KRAS- NRAS- and BRAF mutations stratified by MSI status in localized CC. This dataset represents a big real-world prospective cohort analyzing genetic alterations and their correlations with clinical and histopathological features, as well as prognostic outcomes in this patient population.

In our cohort the prevalence of MSI-H in stage I-III CC was 20.8%. Previous studies have reported MSI-H prevalence in CC ranging from 10% to 20% (14–17). Notably, the distribution of molecular subtypes within our study cohort revealed that 37.7% had All-WT tumors, 39.8% had KRAS mutations, 3% had NRAS mutations, and 19.5% had BRAF mutations. Prior studies have reported varying prevalence rates with KRAS mutations ranging from 28.7% to 49.3%, NRAS mutations from 2.2% to 9%, and BRAF mutations from 4% to 14% (8, 16, 18–28). It is important to acknowledge that previous investigations often focused on specific stages of CC, or included metastatic/recurrent colon and/or rectal cancer, potentially contributing to observed differences. Moreover, some studies narrowly examined specific codon mutations within the KRAS gene, potentially leading to an underrepresentation of mutation frequencies. In contrast, our study identified a broader spectrum of KRAS mutations, with approximately 15% located in codon 146 or other codons, highlighting the complexity of mutational landscapes in CC. In this extensive cohort, we observed a higher prevalence of both All-WT (46.2% vs. 21.1%) and KRAS MT tumors (46.2% vs. 15.4%), and a lower incidence of BRAF MT tumors (8.1% vs. 62.8%) within the MSS population compared to MSI-H CC. Consistent with our findings, a recent study by Taieb et al. demonstrated analogous distinctions between MSS and MSI-H Stage III CC (28). BRAF mutations have been shown to be associated with the MSI-H subtype, as confirmed by our data (29). In terms of clinical and histopathological features, both KRAS and BRAF MT tumors in both MSS and MSI-H subsets exhibited a preference for localization in the right colon, consistent with findings from previous studies (30–32). Supporting this, analysis from the cancer genome atlas dataset revealed that KRAS mutations were present in 45.5% of right-sided and 40.3% of left-sided colorectal cancers, while BRAF mutations occurred in 24.2% and 2.1% of cases, respectively (33). Additionally, BRAF MT tumors in both subsets demonstrated a higher frequency among female pts. Significantly, the vast majority of BRAF mutations were attributed to the well-known BRAF V600E mutation. In the MSS population, both KRAS and BRAF MT tumors were more frequently diagnosed at advanced disease stages, with BRAF mutations being associated with poorer differentiation, findings in line with previous research on CC (20, 24, 28, 30). Notably, BRAF mutations have been linked with more aggressive CC subtypes with a poorer prognosis (18, 34, 35). Conversely, RAS MT tumors in the MSI-H population, although rare, were more prevalent among younger, male pts and exhibited better histological differentiation.

One might speculate whether these differences may be attributed to additional alterations in various genetic pathways. For instance, alterations in the DNA polymerase genes could cause a hypermutated phenotype. In the MSS cohort, the prevalence of POLE and POLD1 mutations shows no correlation with the BRAF/KRAS mutation status. Both KRAS/BRAF mutated and WT cases show a low mutation rate (1-4%) (**Supplementary Table 5**).

In contrast, the MSI cohort shows a higher frequency of pathogenic POLE and POLD1 mutations. POLE mutations were detected in ~6% of KRAS or BRAF mutated pts. and 13.4% in BRAF/KRAS WT cases. POLD1 mutation were even more frequent (KRAS/BRAF MT 20-25%, WT cases 31.7%) (**Supplementary Table 5**). Whether these are primary mutations in the polymerase genes or a secondary effect of deficient mismatch repair needs to be subject of further analysis.

These findings underscore the heterogeneity of clinicopathological characteristics associated with different mutation profiles in CC, emphasizing the importance of molecular profiling in patient stratification and the development of personalized treatment approaches. Double mutations involving RAS and BRAF genes or within one gene were rare and therefore excluded from further analysis in this study. Consistent with existing literature, the majority of KRAS mutations were found in codons 12 and 13 (8), constituting ~ 85% of all KRAS mutations in MSS and ~ 70% in MSI-H subsets. In MSS tumors, the most common KRAS mutations were p.G12D, followed by p.G12V, in line with findings from the COSMIC database, suggesting these mutations are the most frequent in CC (36). Conversely, in the MSI-H subset, p.G13D followed by p.G12D were more prevalent, consistent with previous observations (28). Notably, the targetable mutations p.G12C mutation was identified in 5.88% of KRAS-mutated MSS cases, with a lower prevalence in MSI-H cases (1.92%), in accordance with other research as summarized in (37). The MSI-H population exhibited a broader spectrum of KRAS mutations, including rare variants. A study analyzing cancer specimens >13000 CRC pts also found a wider distribution of KRAS mutations in the MSI-H subset, including ~9% p.A146T and ~22% other missense structural variants (38). These differences underscore the biological distinctiveness of MSS and MSI-H CC and support the need to consider them as distinct entities with potentially differing therapeutic strategies in the future. Given the remarkable success of immune checkpoint inhibitors in treating localized MSI-H CC (39), they may become a standard therapeutic option for this patient category. Furthermore, these differences are noteworthy when considering that codon 12, but not codon 13 mutations, have been linked to resistance to anti-EGFR therapy and poorer prognosis in CC (40–43).

The survival analysis conducted in our cohort revealed a distinct pattern regarding the influence of RAS and BRAF mutations on DFS and OS among MSI-H and MSS pts. RAS and BRAF mutations showed no significant impact on survival among MSI-H pts, who generally exhibited longer median DFS and OS compared to MSS pts (although no statistical comparison between MSS and MSI-H groups was conducted in our analysis). In contrast, BRAF MT pts in the MSS cohort displayed a significantly shorter DFS (HR 1.74) and showed a clear tendency towards worse OS (HR 1.6),. Remarkably, RAS mutations did not emerge as a significant factor affecting the survival outcomes of MSS pts within our heterogeneous cohort. KRAS and BRAF mutations have frequently been associated with poorer survival in metastatic disease (44, 45), however, their impact in localized stages has delivered conflicting results. For instance, a pooled analysis of the PETACC-3, EORTC 40993 and SAKK 60-00 trials demonstrated no major prognostic value of KRAS mutations regarding relapse-free survival (RFS) or OS, while BRAF mutation was not prognostic for RFS, but was for OS, particularly in pts with MSS tumors in this analysis (46). Other studies, including *post-hoc* analysis of large randomized adjuvant trials and meta-analysis have demonstrated detrimental effects of both KRAS (and its subtypes) and BRAF MT on prognostic endpoints not only including DFS and OS but also time to recurrence and survival after recurrence (24, 28, 47).

The conflicting results observed in studies may arise from variations in study populations, methodologies, and treatment modalities. Differences in patient characteristics, such as age, tumor stage, and comorbidities, could confound the interpretation of survival outcomes. Additionally, the inherent biological disparities between MSS and MSI-H tumors may lead to distinct responses to mutational events. MSI-H tumors, characterized by high levels of genomic instability, may trigger immune responses and result in a better prognosis compared to MSS tumors, which are generally more genomically stable (48, 49). This fundamental difference in tumor biology could influence the prognostic significance of RAS and BRAF mutations in each subtype. Importantly, the poor prognosis observed in MSS BRAF MT pts underscores the urgent need for the development of improved therapeutic strategies and tailored treatments for this subset of pts.

However, our study has certain limitations. Firstly, the registry is not based on a random sample, potentially limiting its representativeness for all CC patients. Secondly, the survival analysis could only adjust for a restricted number of factors, leaving room for additional (partially, possibly unknown) factors necessitating adjustment. Furthermore, this study is fundamentally exploratory, thereby precluding definitive confirmatory statements. Nonetheless, these findings highlight the complex interplay between molecular subtypes, clinical and histopathological features, and survival outcomes in CC, emphasizing the necessity for further investigation into the underlying mechanisms driving these associations. Considering these results, stratifying pts according to molecular subtypes may offer advantages in personalized treatment approaches. Given the relatively small numbers of certain mutations and the limited opportunities to analyze pts in randomized trials, real-world data analyzing larger cohorts like ours may play a pivotal role in advancing our understanding of the biological mechanisms driving outcomes for colorectal cancer pts.

## Data Availability

The original contributions presented in the study are included in the article/supplementary material, further inquiries can be directed to the corresponding author.
