# Supplementary material for: Exploring the molecular profile of localized colon cancer: insights from the AIO Colopredict Plus registry
Source: Front Oncol. 2024 Nov 19;14:1434791. doi: 10.3389/fonc.2024.1434791 (PMC11612501; doi:10.3389/fonc.2024.1434791)
Supplement: Supplementary file 1 [file DataSheet1.pdf]

**Suppl. Table 1: Colorectal Cancer Panel**

The following genes were analyzed for base substitutions, indels (small insertions and deletions) and gene copy number variations (amplifications, deletions) using next generation sequencing:
*ACVR1B, AKT1, APC, ATM, ATP6V0D2, AXIN2, BAX, BLM, BMPR1A, BRAF, BRCA1, BRCA2, BUB1B, CASP8, CDC27, CDH1, CDK4, CDKN2A, CHEK2, CTNNA1, CTNNB1, DCC, DMD, EGFR, ENG, EP300, EPCAM, ERBB2, FBXW7, FGFR3, FLCN, FZD3, GALNT12, GPC6, GREM1, KIT, KRAS, MAP2K4, MAP7, MET, MIER3, MLH1, MLH3, MSH2, MSH3, MSH6, MUTYH, MYO1B, NRAS, PALB2, PIK3CA, PIK3R1, PMS1, PMS2, POLD1, POLE, PTEN, PTPN12, RET, RPS20, SCG5, SLC9A9, SMAD2, SMAD4, SRC, STK11, TCERG1, TCF7L2, TGFBR2, TP53, WBSCR17*

**Suppl. Table 2: Clinical and histopathological baseline characteristics of molecular subgroups in the overall population**

BMI: Body Mass Index; BRAF MT: mutated for B-Raf proto-oncogene, serine/threonine kinase; dMMR: deficient mismatch repair; KRAS MT: mutated for Kirsten Rat Sarcoma; MSI-H: microsatellite instability; MSS microsatellite stability; NRAS MT: mutated for Neuroblastoma ras; pMMR: proficient mismatch repair protein; SD: Standard deviation; WT: Wild Type. In this table, 3 patients with missing MSI Status have been included.

| Characteristic | | | | | p-Value |
|---|---|---|---|---|---|
| | All-WT (N=1843) | KRAS MT (N=1946) | NRAS MT (N=146) | BRAF MT (N=951) | |
| **Age (in years)** | | | | | **<0.001** |
| Median | 71 | 71 | 70.5 | 75 | |
| Mean (SD) | 69.2 (12.1) | 69.4 (11.8) | 68 (12.6) | 73.7 (10.3) | |
| Range | 25 - 95 | 27 - 96 | 26- 92 | 29 - 100 | |
| **Age (categories)** | | | | | **<0.001** |
| < 50 | 127 (6.9%) | 103 (5.3%) | 11 (7.5%) | 16 (1.7%) | |
| 50 -70 | 704 (38.2%) | 785 (40.3%) | 57 (39.0%) | 270 (28.4%) | |
| ≥ 70 | 1012 (54.9%) | 1058 (54.4%) | 78 (53.4%) | 665 (69.9%) | |
| **Sex** | | | | | **<0.001** |
| Female | 724 (39.4%) | 870 (44.8%) | 66 (45.2%) | 642 (67.6%) | |
| Male | 1112 (60.6%) | 1072 (55.2%) | 80 (54.8%) | 308 (32.4%) | |
| Missing | 7 | 4 | 0 | 1 | |
| **CCI** | | | | | 0.53 |
| < 5 | 1819 (98.7%) | 1924 (98.9%) | 146 (100.0%) | 938 (98.6%) | |
| ≥ 5 | 24 (1.3%) | 22 (1.1%) | 0 (0.0%) | 13 (1.4%) | |
| **BMI** | | | | | 0.14 |
| Mean (SD) | 26.99 (5.2) | 26.7 (5.0) | 26.5 (4.8) | 26.6 (5.1) | |
| Range | 12.7 - 57.5 | 14.5 - 54.6 | 15.9 - 42.2 | 13.8 - 52.5 | |
| Missing | 140 | 169 | 8 | 92 | |
| **BMI (categories)** | | | | | 0.50 |
| < 18.5 | 47 (2.8%) | 45 (2.5%) | 5 (3.6%) | 24 (2.8%) | |
| 18.5 - 25 | 602 (35.3%) | 689 (38.8%) | 47 (34.1%) | 321 (37.4%) | |
| ≥ 25 | 1054 (61.9%) | 1043 (58.7%) | 86 (62.3%) | 514 (59.8%) | |
| Missing | 140 | 169 | 8 | 92 | |
| **T-Stage** | | | | | 0.45 |
| T 1/2 | 284 (15.4%) | 285 (14.6%) | 16 (11.0%) | 149 (15.7%) | |
| T 3/4 | 1559 (84.6%) | 1661 (85.4%) | 130 (89.0%) | 802 (84.3%) | |
| **N-Stage** | | | | | 0.13 |
| N0 | 1121 (61.1%) | 1128 (58.2%) | 82 (56.2%) | 539 (57.1%) | |
| N+ | 714 (38.9%) | 810 (41.8%) | 64 (43.8%) | 405 (42.9%) | |
| **UICC** | | | | | 0.059 |
| I | 202 (11.0%) | 182 (9.4%) | 7 (4.8%) | 100 (10.5%) | |
| II | 918 (49.8%) | 951 (48.9%) | 76 (52.1%) | 438 (46.1%) | |
| III | 722 (39.2%) | 813 (41.8%) | 63 (43.2%) | 413 (43.4%) | |
| **Differential Grade** | | | | | **<0.001** |
| G 1/2 | 1486 (80.6%) | 1603 (82.4%) | 122 (83.6%) | 535 (56.3%) | |
| G 3/4 | 357 (19.4%) | 343 (17.6%) | 24 (16.4%) | 416 (43.7%) | |
| **Histological Subtype** | | | | | **<0.001** |
| Adenocarcinoma | 1721 (95.5%) | 1806 (94.7%) | 138 (95.8%) | 824 (87.8%) | |
| Mucinous adenocarcinoma | 44 (2.4%) | 77 (4.0%) | 6 (4.2%) | 80 (8.5%) | |
| Signet-ring cell carcinoma | 7 (0.4%) | 0 (0.0%) | 0 (0.0%) | 4 (0.4%) | |
| Others | 30 (1.7%) | 24 (1.3%) | 0 (0.0%) | 30 (3.2%) | |
| Missing | 41 | 39 | 2 | 13 | |
| **Chemotherapy** | | | | | **0.001** |
| Yes | 649 (39.5%) | 723 (41.8%) | 53 (43.1%) | 288 (33.9%) | |
| No | 996 (60.5%) | 1005 (58.2%) | 70 (56.9%) | 561 (66.1%) | |
| Missing | 198 | 218 | 23 | 102 | |
| **Localisation** | | | | | **<0.001** |
| Cecum | 150 (8.2%) | 411 (21.5%) | 22 (15.2%) | 206 (21.8%) | |
| Ascending colon | 335 (18.4%) | 504 (26.3%) | 34 (23.4%) | 401 (42.4%) | |
| Hepatic flexure | 114 (6.3%) | 110 (5.7%) | 5 (3.4%) | 100 (10.6%) | |
| Transverse colon | 194 (10.7%) | 154 (8.0%) | 11 (7.6%) | 106 (11.2%) | |
| Splenic flexure | 70 (3.8%) | 86 (4.5%) | 7 (4.8%) | 26 (2.8%) | |
| Descending colon | 138 (7.6%) | 97 (5.1%) | 11 (7.6%) | 35 (3.7%) | |
| Sigmoid colon | 761 (41.8%) | 519 (27.1%) | 50 (34.5%) | 68 (7.2%) | |
| Rectosigmoid junction | 58 (3.2%) | 33 (1.7%) | 5 (3.4%) | 3 (0.3%) | |
| Missing | 23 | 32 | 1 | 6 | |
| **Sidedness** | | | | | **<0.001** |
| Right | 793 (43.6%) | 1179 (61.6%) | 72 (49.7%) | 813 (86.0%) | |
| Left | 1027 (56.4%) | 735 (38.4%) | 73 (50.3%) | 132 (14.0%) | |
| Missing | 23 | 32 | 1 | 6 | |
| **MSI Status** | | | | | **<0.001** |
| MSS/pMMR | 1627 (88.3%) | 1787 (91.9%) | 139 (95.2%) | 312 (32.8%) | |
| MSI-H/dMMR | 215 (11.7%) | 157 (8.1%) | 7 (4.8%) | 639 (67.2%) | |
| Missing | 1 | 2 | 0 | 0 | |

**Suppl. Table 3: Clinical and histopathological baseline characteristics of distinct KRAS mutations the MSS and MSI- H cohort**

BMI: Body Mass Index; dMMR: deficient mismatch repair; KRAS MT: mutated for Kirsten Rat Sarcoma; MSI-H: microsatellite instability; MSS microsatellite stability; pMMR: proficient mismatch repair protein; SD: Standard deviation; WT: Wild Type

| Characteristic | KRAS MT MSS/pMMR | | | | | | | p-Value | KRAS MT MSI/dMMR | | | | | | | p-Value |
|---|---|---|---|---|---|---|---|---|---|---|---|---|---|---|---|---|
| | p.G12D (N=533) | p.G13D (N=268) | p.G12V (N=373) | p.G12A (N=103) | p.A146T (N=90) | p.G12C (N=106) | Others (N=278) | | p.G12D (N=34) | p.G13D (N=44) | p.G12V (N=10) | p.G12A (N=6) | p.A146T (N=9) | p.G12C (N=3) | other (N=46) | P value |
| **Age (in years)** | | | | | | | | 0.74 | | | | | | | | 0.30 |
| Median | 71 | 70 | 72 | 71 | 72 | 71.5 | 72 | | 61.5 | 65.6 | 68 | 69.5 | 74 | 77 | 71.5 | |
| Mean (SD) | 69.4 (11.5) | 69 (11.6) | 70.3 (11.6) | 70.4 (11.8) | 68.9 (12.9) | 70 (9.8) | 70 (11.3) | | 62 (15.1) | 64.4 (14.3) | 68.5 (13.0) | 67.7 (10.5) | 69 (15.2) | 78.3 (7.1) | 68.3 (13.8) | |
| Range | 31 - 93 | 35 - 93 | 30 - 96 | 33 - 96 | 34 - 92 | 42 - 87 | 37 - 96 | | 29 - 85 | 27 - 90 | 49 - 94 | 51 - 78 | 43 - 90 | 72 - 86 | 29 - 87 | |
| **Age (categories)** | | | | | | | | 0.29 | | | | | | | | 0.58 |
| < 50 | 26 (4.9%) | 12 (4.5%) | 18 (4.8%) | 2 (1.9%) | 9 (10.0%) | 1 (0.9%) | 12 (4.3%) | | 7 (20.6%) | 7 (15.9%) | 1 (10.0%) | 0 (0.0%) | 1 (11.1%) | 0 (0.0%) | 5 (10.9%) | |
| 50 -70 | 220 (41.3%) | 115 (42.9%) | 138 (37.0%) | 42 (40.8%) | 32 (35.6%) | 44 (41.5%) | 114 (41.0%) | | 12 (35.3%) | 20 (45.5%) | 5 (50.0%) | 3 (50.0%) | 2 (22.2%) | 0 (0.0%) | 15 (32.6%) | |
| ≥ 70 | 287 (53.8%) | 141 (52.6%) | 217 (58.2%) | 59 (57.3%) | 49 (54.4%) | 61 (57.5%) | 152 (54.7%) | | 15 (44.1%) | 17 (38.6%) | 4 (40.0%) | 3 (50.0%) | 6 (66.7%) | 3 (100.0%) | 26 (56.5%) | |
| **Sex** | | | | | | | | 0.94 | | | | | | | | **0.048** |
| Female | 247 (46.3%) | 119 (44.4%) | 158 (42.7%) | 44 (42.7%) | 39 (43.3%) | 49 (46.7%) | 121 (43.5%) | | 11 (32.4%) | 18 (40.9%) | 5 (50.0%) | 1 (16.7%) | 5 (55.6%) | 2 (66.7%) | 30 (65.2%) | |
| Male | 286 (53.7%) | 149 (55.6%) | 212 (57.3%) | 59 (57.3%) | 51 (56.7%) | 56 (53.3%) | 157 (56.5%) | | 23 (67.6%) | 26 (59.1%) | 5 (50.0%) | 5 (83.3%) | 4 (44.4%) | 1 (33.3%) | 16 (34.8%) | |
| Missing | 0 | 0 | 3 | 0 | 0 | 1 | 0 | | | | | | | | | |
| **CCI** | | | | | | | | 0.47 | | | | | | | | 0.87 |
| < 5 | 526 (98.7%) | 267 (99.6%) | 367 (98.4%) | 102 (99.0%) | 90 (100.0%) | 106 (100.0%) | 273 (98.2%) | | 34 (100.0%) | 43 (97.7%) | 10 (100.0%) | 6 (100.0%) | 9 (100.0%) | 3 (100.0%) | 46 (100.0%) | |
| ≥ 5 | 7 (1.3%) | 1 (0.4%) | 6 (1.6%) | 1 (1.0%) | 0 (0.0%) | 0 (0.0%) | 5 (1.8%) | | 0 (0.0%) | 1 (2.3%) | 0 (0.0%) | 0 (0.0%) | 0 (0.0%) | 0 (0.0%) | 0 (0.0%) | |
| **BMI** | | | | | | | | 0.70 | | | | | | | | 0.51 |
| Mean (SD) | 26.674 (5.040) | 27 (5.7) | 26.5 (4.8) | 26.5 (4.2) | 25.9 (4.2) | 26.6 (5.0) | 26.7 (5.0) | | 25.1 (4.3) | 27 (3.9) | 25.6 (3.7) | 24.5 (4.4) | 26.9 (2.5) | 27.7 (3.3) | 26.9 (5.4) | |
| Range | 15.060 - 53.830 | 14.5 - 54.2 | 16.5 - 45.3 | 15.8 - 35.5 | 17.7 - 43.5 | 16.2 - 40.7 | 15.4 - 47.6 | | 16.7 - 36.6 | 19.6 - 35.7 | 20.6- 30.9 | 19.5 - 30.4 | 24.1 - 31.0 | 24.2 - 30.8 | 19.8 - 45.2 | |
| Missing | 42 | 24 | 24 | 11 | 6 | 10 | 31 | | 4 | 6 | 0 | 1 | 2 | 0 | 6 | |
| **BMI (categories)** | | | | | | | | 0.92 | | | | | | | | 0.36 |
| < 18.5 | 12 (2.4%) | 4 (1.6%) | 11 (3.2%) | 1 (1.1%) | 2 (2.4%) | 4 (4.2%) | 5 (2.0%) | | 3 (10.0%) | 0 (0.0%) | 0 (0.0%) | 0 (0.0%) | 0 (0.0%) | 0 (0.0%) | 0 (0.0%) | |
| 18.5 - 25 | 193 (39.3%) | 96 (39.3%) | 131 (37.5%) | 38 (41.3%) | 39 (46.4%) | 37 (38.5%) | 95 (38.5%) | | 13 (43.3%) | 11 (28.9%) | 4 (40.0%) | 2 (40.0%) | 3 (42.9%) | 1 (33.3%) | 14 (35.0%) | |
| ≥ 25 | 286 (58.2%) | 144 (59.0%) | 207 (59.3%) | 53 (57.6%) | 43 (51.2%) | 55 (57.3%) | 147 (59.5%) | | 14 (46.7%) | 27 (71.1%) | 6 (60.0%) | 3 (60.0%) | 4 (57.1%) | 2 (66.7%) | 26 (65.0%) | |
| Missing | 42 | 24 | 24 | 11 | 6 | 10 | 31 | | 4 | 6 | 0 | 1 | 2 | 0 | 6 | |
| **T-Stage** | | | | | | | | 0.26 | | | | | | | | 0.19 |
| T 1/2 | 76 (14.3%) | 50 (18.7%) | 49 (13.1%) | 11 (10.7%) | 14 (15.6%) | 20 (18.9%) | 36 (12.9%) | | 2 (5.9%) | 4 (9.1%) | 3 (30.0%) | 0 (0.0%) | 0 (0.0%) | 1 (33.3%) | 5 (10.9%) | |
| T 3/4 | 457 (85.7%) | 218 (81.3%) | 324 (86.9%) | 92 (89.3%) | 76 (84.4%) | 86 (81.1%) | 242 (87.1%) | | 32 (94.1%) | 40 (90.9%) | 7 (70.0%) | 6 (100.0%) | 9 (100.0%) | 2 (66.7%) | 41 (89.1%) | |
| **N-Stage** | | | | | | | | 0.34 | | | | | | | | 0.87 |
| N0 | 311 (58.3%) | 140 (52.8%) | 209 (56.3%) | 54 (52.9%) | 52 (58.4%) | 53 (50.0%) | 169 (60.8%) | | 25 (73.5%) | 34 (77.3%) | 7 (70.0%) | 6 (100.0%) | 7 (77.8%) | 2 (66.7%) | 33 (73.3%) | |
| N+ | 222 (41.7%) | 125 (47.2%) | 162 (43.7%) | 48 (47.1%) | 37 (41.6%) | 53 (50.0%) | 109 (39.2%) | | 9 (26.5%) | 10 (22.7%) | 3 (30.0%) | 0 (0.0%) | 2 (22.2%) | 1 (33.3%) | 12 (26.7%) | |
| **UICC** | | | | | | | | 0.063 | | | | | | | | 0.46 |
| I | 52 (9.8%) | 32 (11.9%) | 35 (9.4%) | 4 (3.9%) | 12 (13.3%) | 12 (11.3%) | 18 (6.5%) | | 2 (5.9%) | 3 (6.8%) | 2 (20.0%) | 0 (0.0%) | 0 (0.0%) | 1 (33.3%) | 2 (4.3%) | |
| II | 259 (48.6%) | 113 (42.2%) | 172 (46.1%) | 48 (46.6%) | 42 (46.7%) | 41 (38.7%) | 151 (54.3%) | | 23 (67.6%) | 32 (72.7%) | 5 (50.0%) | 6 (100.0%) | 7 (77.8%) | 1 (33.3%) | 31 (67.4%) | |
| III | 222 (41.7%) | 123 (45.9%) | 166 (44.5%) | 51 (49.5%) | 36 (40.0%) | 53 (50.0%) | 109 (39.2%) | | 9 (26.5%) | 9 (20.5%) | 3 (30.0%) | 0 (0.0%) | 2 (22.2%) | 1 (33.3%) | 13 (28.3%) | |
| **Differential Grade** | | | | | | | | 0.79 | | | | | | | | 0.74 |
| G 1/2 | 441 (82.7%) | 229 (85.4%) | 315 (84.5%) | 84 (81.6%) | 72 (80.0%) | 86 (81.1%) | 236 (84.9%) | | 23 (67.6%) | 30 (68.2%) | 8 (80.0%) | 5 (83.3%) | 5 (55.6%) | 3 (100.0%) | 30 (65.2%) | |
| G 3/4 | 92 (17.3%) | 39 (14.6%) | 58 (15.5%) | 19 (18.4%) | 18 (20.0%) | 20 (18.9%) | 42 (15.1%) | | 11 (32.4%) | 14 (31.8%) | 2 (20.0%) | 1 (16.7%) | 4 (44.4%) | 0 (0.0%) | 16 (34.8%) | |
| **Histological Subtype** | | | | | | | | 0.12 | | | | | | | | 0.96 |
| Adenocarcinoma | 485 (92.9%) | 251 (95.1%) | 346 (95.8%) | 98 (98.0%) | 85 (94.4%) | 101 (97.1%) | 264 (96.0%) | | 31 (91.2%) | 40 (90.9%) | 9 (100.0%) | 5 (83.3%) | 9 (100.0%) | 3 (100.0%) | 38 (88.4%) | |
| Mucinous adenocarcinoma | 28 (5.4%) | 6 (2.3%) | 12 (3.3%) | 2 (2.0%) | 5 (5.6%) | 3 (2.9%) | 9 (3.3%) | | 2 (5.9%) | 4 (9.1%) | 0 (0.0%) | 1 (16.7%) | 0 (0.0%) | 0 (0.0%) | 4 (9.3%) | |
| Others | 9 (1.7%) | 7 (2.7%) | 3 (0.8%) | 0 (0.0%) | 0 (0.0%) | 0 (0.0%) | 2 (0.7%) | | 1 (2.9%) | 0 (0.0%) | 0 (0.0%) | 0 (0.0%) | 0 (0.0%) | 0 (0.0%) | 1 (2.3%) | |
| Missing | 11 | 4 | 12 | 3 | 0 | 2 | 3 | | 0 | 0 | 1 | 0 | 0 | 0 | 3 | |
| **Chemotherapy** | | | | | | | | 0.46 | | | | | | | | 0.60 |
| Yes | 190 (40.7%) | 116 (48.3%) | 146 (43.8%) | 43 (46.7%) | 32 (40.0%) | 39 (41.5%) | 101 (40.1%) | | 9 (30.0%) | 11 (30.6%) | 0 (0.0%) | 1 (16.7%) | 2 (25.0%) | 1 (33.3%) | 11 (28.2%) | |
| No | 277 (59.3%) | 124 (51.7%) | 187 (56.2%) | 49 (53.3%) | 48 (60.0%) | 55 (58.5%) | 151 (59.9%) | | 21 (70.0%) | 25 (69.4%) | 10 (100.0%) | 5 (83.3%) | 6 (75.0%) | 2 (66.7%) | 28 (71.8%) | |
| Missing | 66 | 28 | 40 | 11 | 10 | 12 | 26 | | 4 | 8 | 0 | 0 | 1 | 0 | 7 | |
| **Localisation** | | | | | | | | **<0.001** | | | | | | | | 0.18 |
| Cecum | 122 (23.2%) | 69 (26.2%) | 69 (18.9%) | 17 (16.7%) | 22 (24.7%) | 19 (18.3%) | 53 (19.4%) | | 4 (12.5%) | 10 (22.7%) | 2 (22.2%) | 2 (33.3%) | 2 (22.2%) | 1 (33.3%) | 9 (19.6%) | |
| Ascending colon | 156 (29.7%) | 59 (22.4%) | 77 (21.1%) | 20 (19.6%) | 29 (32.6%) | 27 (26.0%) | 76 (27.8%) | | 6 (18.8%) | 12 (27.3%) | 3 (33.3%) | 1 (16.7%) | 2 (22.2%) | 2 (66.7%) | 22 (47.8%) | |
| Hepatic flexure | 13 (2.5%) | 18 (6.8%) | 17 (4.7%) | 14 (13.7%) | 4 (4.5%) | 12 (11.5%) | 18 (6.6%) | | 1 (3.1%) | 1 (2.3%) | 1 (11.1%) | 0 (0.0%) | 2 (22.2%) | 0 (0.0%) | 5 (10.9%) | |
| Transverse colon | 50 (9.5%) | 18 (6.8%) | 24 (6.6%) | 10 (9.8%) | 10 (11.2%) | 9 (8.7%) | 15 (5.5%) | | 5 (15.6%) | 4 (9.1%) | 0 (0.0%) | 1 (16.7%) | 2 (22.2%) | 0 (0.0%) | 3 (6.5%) | |
| Splenic flexure | 19 (3.6%) | 10 (3.8%) | 20 (5.5%) | 3 (2.9%) | 7 (7.9%) | 4 (3.8%) | 12 (4.4%) | | 4 (12.5%) | 4 (9.1%) | 0 (0.0%) | 1 (16.7%) | 0 (0.0%) | 0 (0.0%) | 1 (2.2%) | |
| Descending colon | 28 (5.3%) | 11 (4.2%) | 23 (6.3%) | 6 (5.9%) | 1 (1.1%) | 3 (2.9%) | 12 (4.4%) | | 7 (21.9%) | 3 (6.8%) | 0 (0.0%) | 0 (0.0%) | 0 (0.0%) | 0 (0.0%) | 1 (2.2%) | |
| Sigmoid colon | 132 (25.1%) | 74 (28.1%) | 129 (35.3%) | 28 (27.5%) | 16 (18.0%) | 26 (25.0%) | 79 (28.9%) | | 5 (15.6%) | 10 (22.7%) | 3 (33.3%) | 1 (16.7%) | 1 (11.1%) | 0 (0.0%) | 5 (10.9%) | |
| Rectosigmoid junction | 6 (1.1%) | 4 (1.5%) | 6 (1.6%) | 4 (3.9%) | 0 (0.0%) | 4 (3.8%) | 8 (2.9%) | | 0 (0.0%) | 0 (0.0%) | 0 (0.0%) | 0 (0.0%) | 0 (0.0%) | 0 (0.0%) | 0 (0.0%) | |
| Missing | 7 | 5 | 8 | 1 | 1 | 2 | 5 | | 2 | 0 | 1 | 0 | 0 | 0 | 0 | |
| **Sidedness** | | | | | | | | **<0.001** | | | | | | | | **0.020** |
| Right | 341 (64.8%) | 164 (62.4%) | 187 (51.2%) | 61 (59.8%) | 65 (73.0%) | 67 (64.4%) | 162 (59.3%) | | 16 (50.0%) | 27 (61.4%) | 6 (66.7%) | 4 (66.7%) | 8 (88.9%) | 3 (100.0%) | 39 (84.8%) | |
| Left | 185 (35.2%) | 99 (37.6%) | 178 (48.8%) | 41 (40.2%) | 24 (27.0%) | 37 (35.6%) | 111 (40.7%) | | 16 (50.0%) | 17 (38.6%) | 3 (33.3%) | 2 (33.3%) | 1 (11.1%) | 0 (0.0%) | 7 (15.2%) | |
| Missing | 7 | 5 | 8 | 1 | 1 | 2 | 5 | | 2 | 0 | 1 | 0 | 0 | 0 | 0 | |

**Suppl. Table 4: Mutations of MMR Genes in the MSI cohort in relation to BRAF V600 MT**
WT: Wild Type; MT: mutated

| Gene | Level | BRAF V600 WT | | | BRAF V600 MT | | |
|---|---|---|---|---|---|---|---|
| | | N | % | ∑% | N | % | ∑% |
| MLH1 | pathogenic | 59 | 22.6 | 22.6 | 30 | 8.3 | 8.3 |
| | WT | 163 | 73.4 | 100 | 332 | 91.7 | 100 |
| MSH2 | pathogenic | 42 | 18.9 | 18.9 | 23 | 6.3 | 6.3 |
| | WT | 180 | 81.1 | 100 | 339 | 93.7 | 100 |
| PMS2 | pathogenic | 35 | 15.8 | 15.8 | 35 | 9.7 | 9.7 |
| | WT | 187 | 84.2 | 100 | 327 | 90.3 | 100 |
| MSH6 | pathogenic | 48 | 21.6 | 21.6 | 64 | 17.7 | 17.7 |
| | WT | 174 | 78.4 | 100 | 298 | 82.3 | 100 |

**Suppl. Table 5 : Mutations of POLE and POLD1 in relation to KRAS and BRAF status**

WT: Wild Type; MT: mutated; MSI: microsatellite instability; MSS: microsatellite stable; KRAS MT: mutated for Kirsten Rat
POLE: DNA Polymerase Epsilon; POLD1: DNA polymerase delta

| KRAS / BRAF status | Gene | Level | MSI | | | MSS | | |
|---|---|---|---|---|---|---|---|---|
| | | | N | % | Σ% | N | % | Σ% |
| KRAS MT BRAF WT | POLD1 | pathogenic | 16 | 21.9 | 21.9 | 38 | 3.8 | 3.8 |
| | | WT | 57 | 78.1 | 100 | 949 | 96.2 | 100 |
| | POLE | pathogenic | 5 | 6.8 | 6.8 | 11 | 1.1 | 1.1 |
| | | WT | 68 | 93.2 | 100 | 976 | 98.9 | 100 |
| KRAS WT BRAF MT | POLD1 | pathogenic | 97 | 26.6 | 26.6 | 8 | 3.7 | 3.7 |
| | | WT | 267 | 73.3 | 100 | 209 | 96.3 | 100 |
| | POLE | pathogenic | 23 | 6.3 | 6.3 | 3 | 1.4 | 1.4 |
| | | WT | 341 | 93.7 | 100 | 214 | 98.6 | 100 |
| KRAS WT BRAF WT | POLD1 | pathogenic | 45 | 31.7 | 31.7 | 31 | 2.8 | 2.8 |
| | | WT | 97 | 68.3 | 100 | 1094 | 97.2 | 100 |
| | POLE | pathogenic | 19 | 13.4 | 13.4 | 13 | 1.2 | 1.2 |
| | | WT | 123 | 86.6 | 100 | 1112 | 98.8 | 100 |
